# Histone Acetylation Dynamics Integrates Metabolic Activity to Regulate Plant Response to Stress

**DOI:** 10.3389/fpls.2019.01236

**Published:** 2019-10-04

**Authors:** Yongfeng Hu, Yue Lu, Yu Zhao, Dao-Xiu Zhou

**Affiliations:** ^1^College of Bioengineering, Jingchu University of Technology, Jingmen, China; ^2^National Key Laboratory of Crop Genetic Improvement, Huazhong Agricultural University, Wuhan, China; ^3^Institute of Plant Science of Paris-Saclay (IPS2), CNRS, INRA, University Paris-sud 11, University Paris-Saclay, Orsay, France

**Keywords:** plant, stress response, histone acetylation, histone acylation, acetyl-CoA, metabolism

## Abstract

Histone lysine acetylation is an essential chromatin modification for epigenetic regulation of gene expression during plant response to stress. On the other hand, enzymes involved in histone acetylation homeostasis require primary metabolites as substrates or cofactors whose levels are greatly influenced by stress and growth conditions in plants. In addition, histone lysine acylation that requires similar enzymes for deposition and removal as histone acetylation has been recently characterized in plant. Results on understanding the intrinsic relationship between histone acetylation/acylation, metabolism and stress response in plants are accumulating. In this review, we summarize recent advance in the field and propose a model of interplay between metabolism and epigenetic regulation of genes expression in plant adaptation to stress.

## Introduction

Due to their sessile life style, plants have developed sophisticated signaling pathways by adjusting gene expression, metabolism, cellular activity, and growth to respond to the changing environmental conditions. During the last 2 decades, much progress has been made in deciphering signaling pathways of plant responses to both biotic and abiotic stresses. More recent data indicate that chromatin modification plays an essential role in epigenetic regulation of gene expression and plant adaptation to stress (Kim et al., 2015). Chromatin modifications, including DNA methylation, histone modifications, histone variant deposition, and nucleosome positioning, are the basis of epigenetic regulation. Nucleosome, which is the building block of chromatin, consists of a core histone octamer (H2A, H2B, H3, and H4) and a segment of DNA wrapped around the core. The linker histone H1 binds to the nucleosome at the entry and exit sites of DNA and is involved in the stability of higher-order structure of chromatin. N-terminal tails of core histones are modified with various chemical groups such as methyl, acetyl, acyl, ribosyl, phosphate as well as ubiquitin and SUMO. Histone modification affects nucleosome and chromatin structure, thus plays important roles in fine tuning of gene expression. Histone modification, in particular histone lysine acetylation, has been shown to play an essential role in plant response and tolerance to stress (Servet et al., 2010; Yuan et al., 2013). In this review we focus on summarizing recent progresses in the field and discussing histone acetylation dynamics and its relationship with primary metabolism in plant responses to stress.

### Histone Acetylation Dynamics in Plant Responses to Stress

Histone acetylation consists of addition of acetyl group, which is provided by acetyl-CoA, to the ε-amine group of N-terminal lysine residues. Histone lysine acetylation not only neutralizes the positive charge of the amine group, and enhances the hydrophobicity and increases the size of the lysine side chain, but also provides platforms for binding by regulatory proteins of chromatin structure and gene activity to promote genes transcription.

Global change in histone acetylation in response to stress has been reported in various plants. For example, salt, drought, and heat stress increase acetylation of histone H3 lysine 9 (H3K9ac) and histone H4 lysine 5 (H4K5ac) levels in different tissues of maize (Li et al., 2014; Zhao et al., 2014a; Zhao et al., 2014b; Wang et al., 2015). In rice seedlings, drought stress induces H3K9ac, H3K18ac, H3K27ac, H3ac, and H4K5ac (Fang et al., 2014). However, cold stress dramatically reduces global H3K14ac while without altering H3K9ac and H3K27ac in the same plant (Roy et al., 2014). Recent results indicate that in rice plants under submergence or starvation, genome-wide H3K9ac displays dynamic changes which correlates with the stress-induced gene activation (Lu et al., 2018). These data illustrate that histone H3 and H4 lysine acetylation is mostly elevated during plant response to stress. The increase implies that either higher acetylation or lower deacetylation activities are induced during stress responses. The global change of acetylation at specific lysine residues suggests that activity of particular enzymes involved in histone acetylation is induced by the stress. However, despite that acetylation enzymes may have preferential lysine targets in histone H3 or H4 (Henry et al., 2013; Kuo and Andrews, 2013; Zhou et al., 2017), acetylation at a particular site can be spread to neighboring lysine residues (Earley et al., 2007). On the other hand, as primary metabolism is greatly altered by stress or growth conditions, a metabolic control of histone acetylation is not excluded (see below). It is generally thought that the increase of histone acetylation plays an essential role in reprograming gene expression for plant response or tolerance to stress. However, the increase of histone acetylation at some loci may lag behind gene activation, thus appears in this case as the consequence rather than the cause of the stress-induced gene expression.

### Function of Histone Acetylation Enzymes in Plant Response to Stress

Histone lysine acetylation is catalyzed by histone acetyltransferases (HATs) and reversed by histone deacetylases (HDACs). In plants, the HATs can be grouped into four classes: General control nondepressible 5 (GCN5)-related Acetyl Transferase (GNAT), MOZ-YBF2/SAS3-SAS2/TIP60 (MYST), cAMP-responsive element Binding Protein (CBP), and TATA-binding protein Associated Factor 1 (TAF1) (Pandey et al., 2002). Histone acetylation at different lysine residues is carried out by specific HATs (Earley et al., 2007). For example, two GNAT class HATs, HAG1 and HAG2, catalyze H3K14 and H4K12 acetylation respectively. Two MYST class HATs, HAM1 and HAM2, redundantly acetylate H4K5. However, multiple HATs are responsible for acetylation of H3K9, H4K8, and H4K16 (Earley et al., 2007). Enzymatic specificities of HATs may reflect their specific roles in the regulation of gene expression, as recognition of different acetylated lysines of histone by distinct reader proteins incur various epigenetic consequences. These proteins include chromatin remodelers, BET proteins, histone lysine methyltransferases and HATs themselves, all of which contain bromodomain that mediates the interaction between the reader protein and acetylated lysine (Marmorstein and Zhou, 2014). Regulatory function of some of these proteins in plant has been characterized. An *Arabidopsis* ATP-dependent chromatin remodeler, BRAHMA (BRM), is required for the expression of many genes in concert with or counteracting histone variant H2A.Z (Han et al., 2015; Torres and Deal, 2019). The bromodomain of BRM is able to bind histones but its targeting acetylated lysine has not been identified yet (Farrona et al., 2007). The HDACs can be grouped into three families: Reduced Potassium Dependency 3 (RDP3)/Histone DeAcetylase 1 (HDA1), Silent Information Regulator 2 (SIR2), and the plant-specific Histone Deacetylase 2 (HD2) (Pandey et al., 2002). In addition, based on their homology to yeast HDACs the plant RPD3/HDA1 family is divided into three classes (I, II, and IV) and their activity can be suppressed by different HDAC inhibitors respectively (Ueda et al., 2017). Site specificity of HDACs on histones has not been clearly demonstrated. Alteration of rice *HDT701* expression level affects global H4K5 and H4K16 acetylation levels, while many HDACs regulates H3 acetylation at specific genes (Ding et al., 2012; Zheng et al., 2016; Liu et al., 2017; Ueda et al., 2017; Wang et al., 2017; Cheng et al., 2018; Park et al., 2018). In addition to histones, non-histone substrates of plant RPD3/HDA1 family HDACs has also been identified (Hartl et al., 2017). These proteins localize either in nucleus or other organelles. The lysine acetylation sites regulated by HDACs account for 10% of total detected lysine acetylation sites (Hartl et al., 2017). These results indicate that HDACs have additional cellular functions aside from regulation of gene expression *via* histone deacetylation. HAT and HDAC proteins target specific genomic or chromatin regions by interacting with transcription factors and other chromatin proteins for epigenetic regulation of gene expression in response to cellular signals (Servet et al., 2010; Liu et al., 2014). It is also possible that the interactions mediate reversible acetylation of transcription regulators by HATs and HDACs, which is important for the function of the regulators. The mechanism has been testified in mammals but not reported in plant yet (Lee et al., 2012; Sadler et al., 2015). Besides, in rice, moonlight function of Glyceraldehyde-3-phosphate dehydrogenase (GAPDH) is modulated by SIR2 family HDAC, SRT1, which will be discussed later. Function of HATs and HDACs in stress-responses was summarized in the previous review (Kim et al., 2015). Here we discuss recent advances in functional analysis of HATs and HDACs in regulating plant response to stress.

GCN5 is a primary histone acetyltransferase in regulating plant gene expression (Servet et al., 2010; Zhou et al., 2017). Recent studies demonstrated a primary role of GCN5 in plant response and tolerance to stress. Mutation or downregulation of *GCN5* increases plant sensitivity to varieties of stresses including salt, drought, heat, and disease in different species (Hu et al., 2015; Kong et al., 2017; Zheng et al., 2018; Li et al., 2019). In *Arabidopsis*, GCN5-mediated salt and heat tolerance is dependent on its regulatory function on stress genes expression (Hu et al., 2015; Zheng et al., 2018). Induction of stress genes is attenuated in *gcn5* mutants and overexpression of the stress genes in *gcn5* mutant plants can partially restore stress tolerance. In *Populus trichocarpa*, GCN5 is recruited to promoters of the drought-responsive genes *PtrNAC006*, *PtrNAC007*, and *PtrNAC120* to activate their expression, thus conferring drought tolerance (Li et al., 2019). In soybean, GCN5-mediated histone acetylation at defense-related genes is important for their activation and plant immunity (Kong et al., 2017). The cytoplasmic effector PsAvh23 produced by the soybean pathogen suppresses histone acetylation by disassembling HAT complex to increase plant susceptibility. The role of the other HATs in plant stress response has not been explored except that HAC1 and HAC5 (HAC1/5) are shown to be essential for salicylic acid (SA) -triggered immunity (Jin et al., 2018). HAC1/5 form a complex with NPR1, which is recruited to *PR* chromatin by TGACG BINDING FACTORs (TGAs) to induce *PR* transcription. HAC1/5 mutation diminishes *PR* induction and plant resistance to pathogen.

Several HDACs have been reported to negatively regulate stress tolerance in *Arabidopsis* such as HDA9, HDA15, HD2C, AtSRT1, Class I HDAC (HDA19) in Col-0 ecotype, and HDA6 in Ler ecotype (Buszewicz et al., 2016; Mehdi et al., 2016; Zheng et al., 2016; Liu et al., 2017; Ueda et al., 2017; Wang et al., 2017; Park et al., 2018; Ueda et al., 2018; Shen et al., 2019). Consistent with the discovery, treatment with HDACs inhibitor Cyclo(-L-2-amino-8-hydroxamido-suberoyl-aminoisobutylyl-L-phenylalanyl-D-prolyl-), called Ky-2, increases plant tolerance to salt stress, which is dependent on enhanced Na^+^ efflux by activation of *AtSOS1* genes (Sako et al., 2016). Defects in *AtSOS1* neutralize salt tolerance caused by ky2 treatment. The phenomenon has also been observed in cassava. Treatment with HDAC inhibitor, suberoylanilide hydroxamic acid (SAHA) induces *MeSOS1* expression and reduces Na^+^ content thus enhancing tolerance to salinity stress in cassava (Patanun et al., 2016). However, other HDACs were found to act as positive regulators of stress tolerance. These include HD2C, HDA19 in Ws ecotype, HDA6 in Ws, and Col-0 ecotype, HD2B and Class II HDACs (HDA5/14/15/18) (Chen et al., 2010; Chen and Wu, 2010; Luo et al., 2012; Latrasse et al., 2017; Ueda et al., 2017). Surprisingly, the same genes in different ecotype appear to function oppositely in stress tolerance. The cause of this functional divergence is unclear. It is suggested that the functional discrepancy between Class I and Class II HDACs may be because they have distinct downstream target genes (Ueda et al., 2017). For instance, there are few common up-regulated genes in *hda19* and *hda5/14/15/18* mutants before and after salt treatment (Ueda et al., 2017). Moreover, stress tolerance-related and stress sensitivity-related genes are respectively activated in *hda19* and *hda5/14/15/18* mutants, suggesting mutation of two classes HDACs induces opposite responses to salt stress. There is also possibility that Class II HDACs target non-histone proteins involved in salt tolerance as global histone acetylation remains unchanged in the mutant. Alternatively, HDA19 may act downstream to Class II HDACs because *hda5/14/15/18/19* mutants exhibit similar salt tolerance as *hda19* (Ueda et al., 2017). To dissect specific function of the different HDACs in stress tolerance, genome-wide identification of their target genes and analysis of changes in histone acetylation at these genes in response to stress are required. In addition, how HDACs respond to stress signaling to control histone acetylation and expression of specific genes remains unclear. The gene or locus specificity of HDACs is at least partly determined by interacting with other partner proteins. It was shown that HDA19 interacts with transcriptional co-repressors such as TOPLESS (TPL), SWI-INDEPENDENT3 (SIN3), and SIN3-LIKE (SNL) proteins and transcription factors such as AP2/EREBP, BES1, SCR-like15, WOX5, etc. to repress genes involved in different developmental and stress-responsive pathways (Song et al., 2005; Long et al., 2006; Krogan et al., 2012; Wang et al., 2013; Ryu et al., 2014; Gao et al., 2015; Pi et al., 2015; Mehdi et al., 2016). HDA9 interacts with POWERDRESS (PWR), a SANT-domain containing protein to regulate heat stress response (Chen et al., 2016; Tasset et al., 2018). HDA15 interacts with the transcription factor HFR1 (long Hypocotyl in Far Red1) to repress warm-temperature response (Shen et al., 2019)

It has been shown that redox modification of HDAC is involved in stress signaling in mammalian cells. In humans, oxidative stress reduces HDAC2 activity by tyrosine nitration, resulting in histone hyperacetylation at the target genes and their activation (Ito et al., 2004). Additionally, in mammals activity of several HDACs is regulated by S-nitrosylation (Nott et al., 2008; Feng et al., 2011; Okuda et al., 2015). In *Arabidopsis*, treatment with the physiological NO donor S-nitrosoglutathione (GSNO) increases global histone acetylation, which is triggered by inhibition of cellular overall HDAC activity (Mengel et al., 2017). Stress responsive genes are activated owing to increased histone acetylation. Moreover, SA induced abundance of histone acetylation is diminished in the presence of the NO scavenger (Mengel et al., 2017). This indicates that histone hyperacetylation induced by SA is dependent on NO production. However, whether inhibition of HDAC activity by NO production involves direct S-nitrosylation of HDAC proteins has not been demonstrated yet. It is hypothesized that under normal conditions, HDACs maintain repressive chromatin state of stress-responsive genes by histone deacetylation to keep gene transcripts at low levels. SA or stress induces NO accumulation, which subsequently inhibits HDAC activity by redox modification of the proteins. This leads to histone hyperacetylation and activation of stress-responsive genes expression.

### Acetyl-CoA Controlled Histone Acetylation in Plant Response to Stress

Histone acetylation consumes acetyl-CoA, which is a precursor for amino acids, lipids, and many secondary metabolites required for plant growth and defense. In addition, acetyl-CoA drives the tricarboxylic acid (TCA) cycle for the production of ATP under aerobic conditions. Acetyl-CoA can be produced by beta-oxidation of fatty acids in peroxisome or by oxidizing pyruvate by the pyruvate dehydrogenase complex (PDC) and can be synthesized by ATP-citrate lyase (ACL) in the cytoplasm and nucleus. Thus, acetyl-CoA level reflects cellular energy status. Recent data firmly demonstrates that histone lysine acetylation is regulated by acetyl-CoA availability. In yeast, unusually high levels of acetyl-CoA have a determinative role in histone acetylation and epigenetic regulation of gene expression (Cai et al., 2011; Shi and Tu, 2013). In yeast or mammals, growth-related genes or genes involved in glucose metabolism are up-regulated by histone hyperacetylation in response to growth factor stimulation or high glucose availability (Etchegaray and Mostoslavsky, 2016). In mammalian cells, up-regulation of these genes relies on ACL that converts glucose-derived citrate into acetyl-CoA in the nucleus (Wellen et al., 2009). Another source of nucleic acetyl-CoA is conversion of acetate by acylCoA synthetase short chain family member 2 (ACSS2). ACSS2-mediated acetyl-CoA production is crucial for histone acetylation as decrease in ACSS2 causes lower level of acetyl-CoA and histone acetylation (Bulusu et al., 2017). Nuclear-localized PDC that generates acetyl-CoA from pyruvate is also required for histone acetylation at specific lysine residues important for expression of cell cycle genes in response to growth factor (Sutendra et al., 2014). Lipid-derived acetyl-CoA promotes histone acetylation at certain lysine residues to activate lipid metabolic genes expression in response to accumulated lipid (Mcdonnell et al., 2016). It is intriguing to disclose how acetyl-CoA originated from distinct precursors promotes acetylation of specific lysine residues of histones and expression of specific genes. Most of enzymes responsible for generation of acetyl-CoA such as ACL, ACSS2 and PDC has been reported to localize in nucleus (Wellen et al., 2009; Sutendra et al., 2014; Li et al., 2017). Additionally, ACSS2 can be recruited to chromatin by interacting with a specific transcription factor (Li et al., 2017). Physical association between ACSS2 and CREB-binding protein (CBP), a histone acetytransferase, has been observed indicating that acetyl-CoA is provided locally to activate histone acetyltransferase that targets specific genes for histone acetylation (Mews et al., 2017). ACL and GCN5 may function in the same pathway to promote histone acetylation despite direct interaction of the two enzymes is not examined (Wellen et al., 2009). These results demonstrate that the acetyl-CoA producing enzymes form complexes with different HATs to enhance acetylation at specific histone lysine residues.

In *Arabidopsis*, it was shown that increased level of acetyl-CoA, caused by mutation of *cytosolic acetyl-CoA carboxylase1* (*ACC1*), predominantly promotes histone hyperacetylation at H3K27 (Chen et al., 2017). Conversely, loss-of-function of *adenosine triphosphate (ATP)-citrate lyase subunit A* (*ACLA*) reduces H3K27ac, supporting a causal relationship between high levels of acetyl-CoA and H3K27ac elevation. It was also observed that increase of H3K27ac at a sub set of genes in *acc1* mutant was dependent on GCN5 (Chen et al., 2017). Transcriptome and metabolome analyses indicate that primary metabolism including amino acid biosynthesis is affected in *acc1* (Chen et al., 2017), demonstrating that in plants acetyl-CoA also connects metabolic state with epigenetic regulation of gene expression. A recent study showed that the fatty acid β-oxidation pathway enzymes (acyl-CoA oxidase 4, multifunctional protein 2, and 3-ketoacyl-CoA thiolase-2) are required for histone acetylation and anti-silencing at some endogenous transgenic loci in *Arabidopsis* (Wang et al., 2019). The finding confirms that histone acetylation can be regulated by acetyl-CoA availability in plant cells and indicate that acetyl-CoA produced from β-oxidation in peroxisome participates in histone modification in the nucleus.

**Figure 1:**
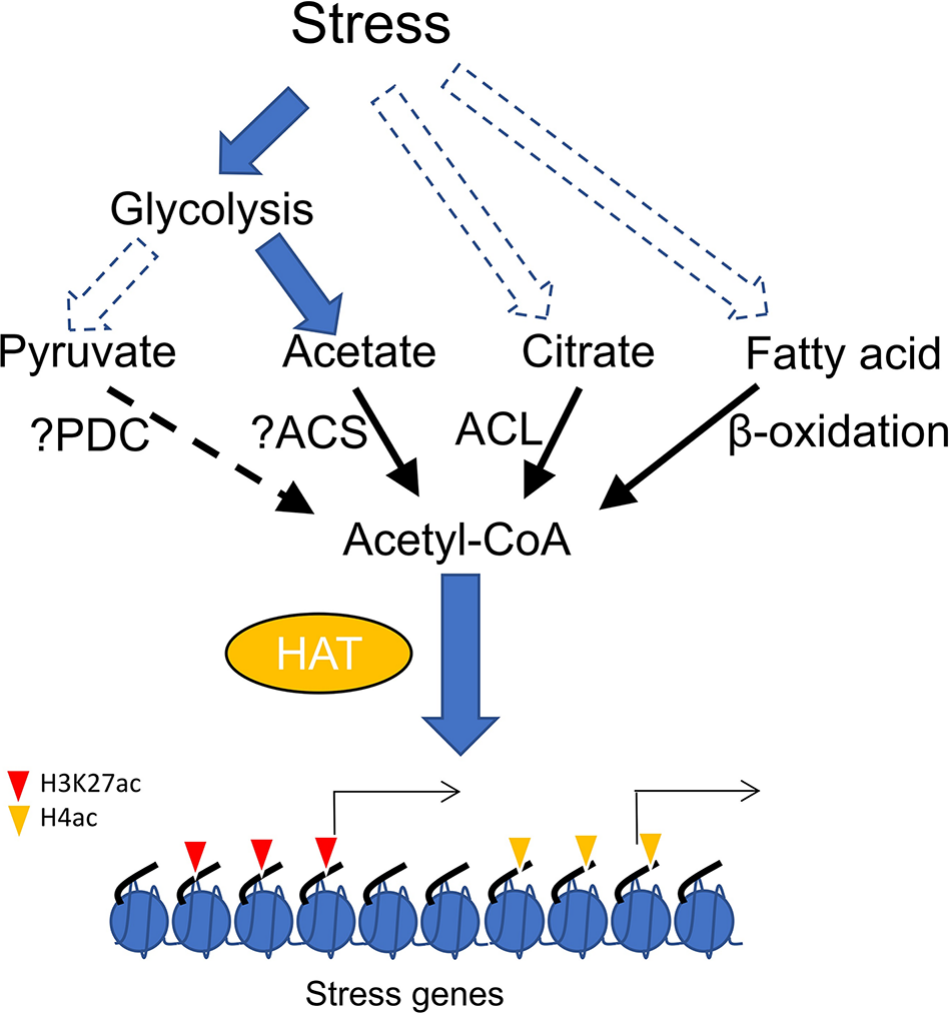
Accumulation of acetyl-CoA stimulates histone acetylation and gene expression in plants, synthesis of acetyl-CoA from different sources including acetate, citrate and fatty acid can be induced by stress. elevation of acetyl-CoA from different sources (citrate and acetate) may stimulate distinct histone acetyl transferases (HAT) to promote histone acetylation at different histone lysine residues (H3K27 and H4) and stress responsive gene expression. PDC, pyruvate dehydrogenase complex; ACS, acetyl-coenzyme A (CoA) synthetase; ACL, ATP-citrate lyase.

Direct evidence supporting how HAT-mediated histone acetylation promoted by acetyl-CoA is involved in stress response has not yet been clearly demonstrated. Plant needs to reduce growth rate to survive from stress conditions, which involves carbon flux from primary metabolism to secondary metabolites (Caretto et al., 2015). The metabolic transition may trigger acetyl-CoA accumulation to enhance global histone acetylation. In addition, glycolysis is generally induced during plant response to stress (Mutuku and Nose, 2012; Lee et al., 2014; Henry et al., 2015), which raises the possibility that pyruvate produced by high glycolytic activity may increase the acetyl-CoA pool. However, it was observed that drought stress induces metabolic flux conversion from glycolysis into acetate synthesis in *Arabidopsis* (Kim et al., 2017). It was shown that application of exogenous acetic acid enhances stress tolerance by promoting histone H4 acetylation and stimulating jasmonic acid (JA) pathway (Kim et al., 2017). Acetate treatment can also increase stress tolerance in other plants, suggesting a general function of acetate metabolism in stress adaptation (Kim et al., 2017). It can be hypothesized that stress induces accumulation of acetic acid which can be converted to acetyl-CoA to enhance histone acetylation (**Figure 1**). Nevertheless, more evidence is required to show whether acetate-promoted histone acetylation is mediated by increased levels of acetyl-CoA. Homolog of ACSS2 in *Arabidopsis* (ACS) catalyzes acetyl-CoA formation in plastid (Lin and Oliver, 2008). Whether this enzyme catalyzes the same reaction in cytosol or nucleus has not yet been demonstrated. Similarly, it remains to show whether *ACS* loss-of-function lowers acetyl-CoA content and histone acetylation level and affects stress tolerance. The induction of H3K27ac by elevated acetyl-CoA in the *acc1* mutant may also play a significant role in stress response, as many genes involved in stress tolerance such as ROS homeostasis, JA response and flavonoid metabolism are up-regulated in the mutant (Chen et al., 2017). However, increased acetyl-CoA from different sources (citrate and acetate) apparently targeted different histone lysine residues (Chen et al., 2017; Kim et al., 2017). One possible explanation may be that specific HATs interact with different enzymatic complexes of acetyl-CoA biosynthesis as described above in animal cells. It is of great interest to uncover how plant histone acetyltransferases discriminate citrate-derived acetyl-CoA from acetate-derived acetyl-CoA to acetylate specific histone lysine residues in response to stress signals. In addition, exploring whether histone acetylation and stress signaling require acetyl-CoA generated from pyruvate and β-oxidation is equally of great importance (**Figure 1**).

### NAD^+^ -Dependent HDAC in Coordinating Metabolism and Stress Response

The sirtuin (SIR2/SIRT)-like proteins require NAD^+^ to remove acetyl from proteins including histones, suggesting that the activity of sirtuins may be controlled by NAD^+^ biosynthesis and consumption. NAD^+^ consumption is likely to be essential in plant response to stress conditions. Downregulation of *poly(ADP*‐*ribose) polymerase (PARP)*, which encodes enzyme transferring ADP-ribose units from NAD^+^ to target proteins, enhances stress tolerance (De Block et al., 2005). However, overexpression of *NADK* encoding enzyme responsible for catalyzing NAD(H) and ATP to produce NADP(H) confers stress tolerance (Li et al., 2018a). Indeed, NADH/NAD^+^ ratio increases while NAD^+^ level is not affected in response to stress (Wang et al., 2016). It is postulated that sirtuins may act as a sensor of cellular redox state to modulate histone acetylation and regulate gene expression in plants (Shen et al., 2016). However, there is still no direct evidence showing that NAD^+^ fluctuation actually regulates SRT1 or SRT2 function in plants.

**Figure 2:**
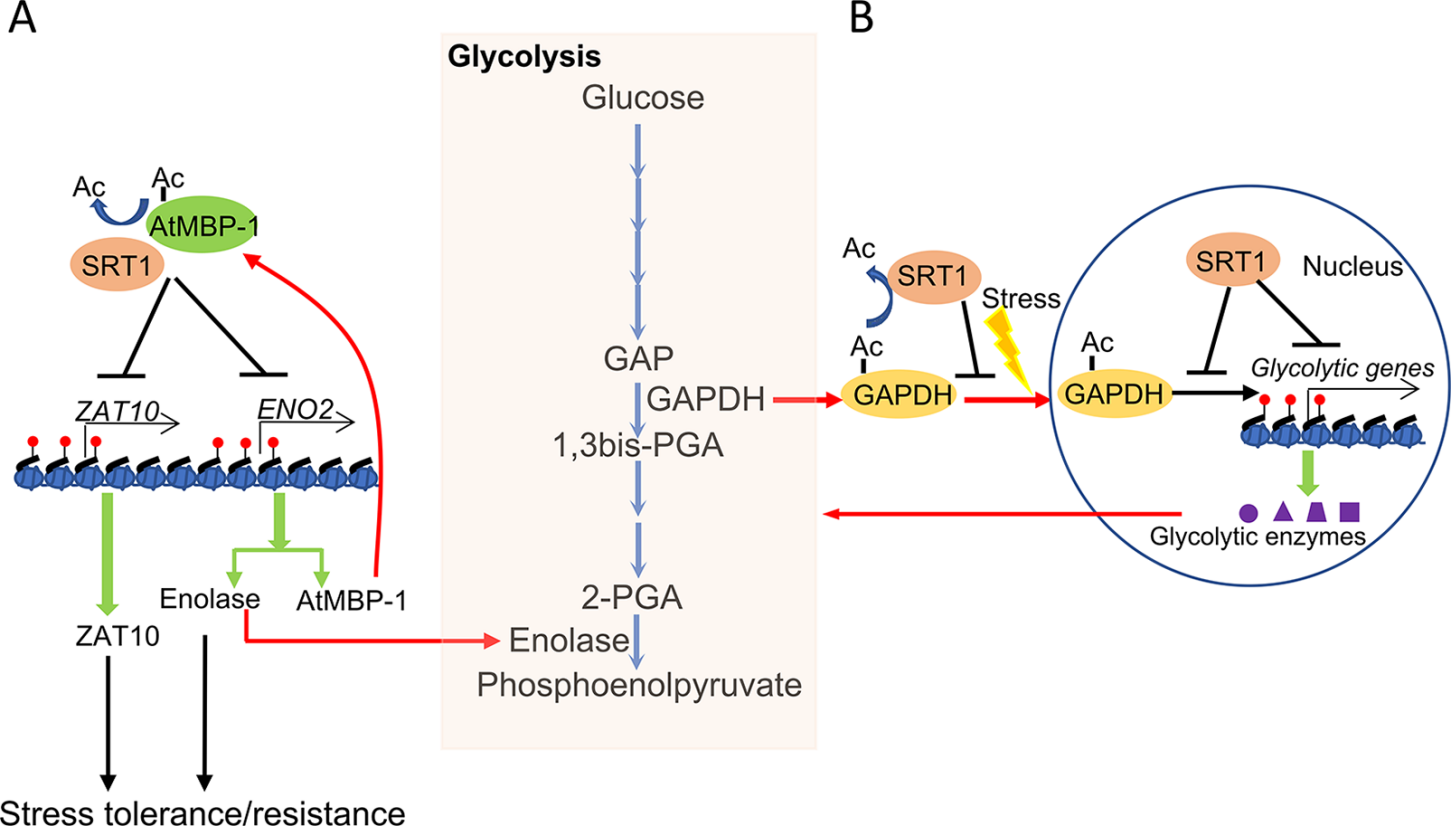
Coordinating metabolism and stress response by plant NAD^+^-dependent histone deacetylase SRT1. **(A)** *Arabidopsis* SRT1 deacetylates and stabilizes AtMBP-1, a direct transcriptional repressor of both stress master regulatory genes ZAT10 and ENO2. ENO2 encodes the glycolytic enolase and AtMBP-1, which are alternative translation products of the ENO2 mRNA and are required for stress tolerance. **(B)** Rice SRT1 represses glycolytic genes expression not only by histone deacetylation of glycolytic genes but also by inhibition of nuclear localization and transcriptional activity of GAPDH through lysine deacetylation.

Glycolysis is enhanced in plants undergoing stress responses (Mutuku and Nose, 2012; Lee et al., 2014; Henry et al., 2015). Interestingly, several glycolytic enzymes also display transcriptional regulatory activities, which are referred to as the moonlighting function of the metabolic enzymes (Boukouris et al., 2016). It was shown that plant SRT1 negatively regulates glycolysis under stress conditions. SRT1 is found to not only deacetylate histones in chromatin of glycolytic genes and reduces the enzymatic activities but to also regulate the moonlighting function of glycolytic enzymes involved in glycolytic gene expression (Liu et al., 2017; Zhang et al., 2017). It was shown that *Arabidopsis* SRT1 deacetylates and stabilizes *Arabidopsis* cMyc-Binding Protein 1 (AtMBP-1), a direct transcriptional repressor of both stress master regulatory genes *STZ*/*ZAT10* and *LOS2*/*ENO2*. The latter encodes glycolytic enolase and AtMBP-1 itself which is in fact produced from an alternative translational initiation of the *LOS2*/*ENO2* mRNA (Kang et al., 2013; Eremina et al., 2015; Liu et al., 2017). *LOS2* is also required for stress tolerance which may be intrinsically related to the alternatively translated AtMBP-1 (Lee et al., 2002; Barkla et al., 2009; Kang et al., 2013). In rice, OsSRT1 represses glycolytic genes expression not only by histone deacetylation of glycolytic genes but also by inhibition of nuclear localization and transcriptional activity of Glyceraldehyde-3-phosphate dehydrogenase (GAPDH) through lysine deacetylation (Zhang et al., 2017). As a rate-limiting enzyme of glycolysis, GAPDH also functions as a transcriptional activator of glycolytic genes under stress, which enhances GAPDH lysine acetylation, nuclear accumulation, and transcriptional activity. These results indicate that SRT1 has a function to coordinate plant sensitivity to abiotic stress and energy metabolism (**Figure 2**).

As high glycolysis leads to a low NAD^+^/NADH ratio in the cell, which may eventually inhibit SRT1 activity, it can be suggested that under stress conditions, enhanced glycolysis in turn inhibits SRT1 and consequently reinforces plant stress tolerance or resistance. Thus, SRT1 may play an important role to regulate carbon metabolic flux of the trade-off between growth and stress tolerance, which is essential for plant adaptation to the changing environment (Shen et al., 2016).

### Histone Acylation and Stress-Response

**Figure 3:**
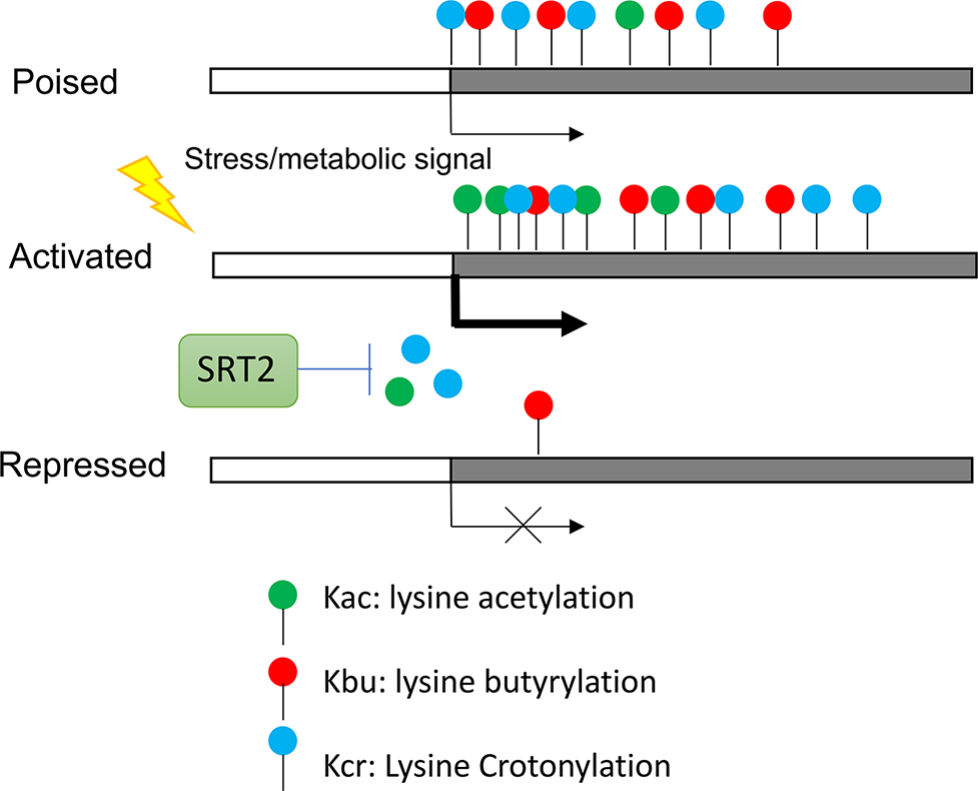
Regulation of stress genes by synergistic effect of histone acetylation and acylation. Genes poised for activation by stress are marked by Kbu and Kcr. Genes activated by stress require histone acetylation. Kcr is removed by NAD^+^ -dependent histone deacetylase SRT2.

Recent results indicate that histones also are acylated by short-chain fatty acids including butyrate, crotonate, 2hydroxyisobutyrate, succinate, malonate, glutarate, etc. (Chen et al., 2007; Tan et al., 2011; Xie et al., 2012). These modifications are similar to lysine acetylation but are distinct in hydrocarbon chain length, hydrophobicity or charge. Studies in animal cells suggest that these histone acylations affect gene expression and are functionally different from histone acetylation (Sabari et al., 2017). In rice plants, most of histone butyrylation (Kbu) and crotonylation (Kcr) sites were shown to overlap with histone acetylation (H3K9ac) sites in highly expressed genes, but subsets of genes show only Kbu and/or Kcr modifications (Lu et al., 2018). The latter genes are most under expressed and stress-inducible. In rice plants under starvation and submergence Kbu and Kcr appear to be less dynamic than H3K9ac and differential changes of the three marks in different sets of genes were observed (**Figure 3**). Thus, the proportion of histone lysine acetylation (H3K9ac) and acylation (Kbu and Kcr) is regulated by stress, suggesting that environmental and metabolic cues control proportional mixture of histone acetylation and acylations in plants, which has functional consequence in chromatin modification and gene expression. The relative levels between histone acetylation and acylations may be dependent on availability of acetyl-CoA and the short fatty acid pools, as same HATs (i.e. P300 in animal cells) can undergo both acetylation and acylation of histones. The reduction of the cytoplasmic and nuclear pools of acetyl-CoA led to an increase in p300-catalysed Kcr in animal cells and the increase in Kcr could be reduced by replenishing the acetyl-CoA pools (Sabari et al., 2015). Thus, under conditions in which acetyl-CoA is reduced, other acyl-CoA forms will be used more often to modify histones. Additionally, it was showed that histone Kcr could be removed by the NAD^+^ -dependent histone deacetylase SRT2 to repress gene expression in rice (Lu et al., 2018) (**Figure 3**). These results indicate that histone acetylation and acylation are at the nexus of the interplay between metabolism, epigenetics, and stress response in plants.

## Conclusion

Plant stress-response signaling pathways have been extensively studied. Under stress, plants have to adjust metabolic activities in favor of stress tolerance. Whether and how the altered metabolic activities are linked to gene regulation are still open questions. Recent findings demonstrating that stress induces metabolic conversion to enhance acetyl-CoA supply for histone acetylation or changes of cellular redox states affecting HDAC activities, establish a link between metabolism, epigenetics, and stress response in plants. In addition, the proportion of acetyl-CoA and different forms of acyl-CoA which can be altered by environmental and metabolic changes is also a significant factor in fine-tuning of stress genes expression. Thus, further understanding the precise mechanisms by which HATs and HDACs activities are modulated by metabolic and redox changes will provide new insight into the complex network regulating plant adaptation and tolerance to stress.

## Author Contributions

YH and D-XZ wrote the paper. YH and YL prepared the figures. YZ modified the paper.

## Funding

This work was supported by grants from the National Key Research and Development Program of China [2016YFD0100802], the National Natural Science Foundation of China [31730049], the Outstanding Young and Middle-aged Science and Technology Innovation Team Project in Higher Education Institutions of Hubei Province [T201621].

## Conflict of Interest

The authors declare that the research was conducted in the absence of any commercial or financial relationships that could be construed as a potential conflict of interest.

## References

[B1] Barkla B. J. Vera-Estrella R. Hernandez-Coronado M. Pantoja O. (2009). Quantitative proteomics of the tonoplast reveals a role for glycolytic enzymes in salt tolerance. Plant Cell 21, 4044–4058. 10.1105/tpc.109.069211 20028841 PMC2814500

[B2] Boukouris A. E. Zervopoulos S. D. Michelakis E. D. (2016). Metabolic enzymes moonlighting in the nucleus: metabolic regulation of gene transcription. Trends Biochem. Sci. 41, 712–730. 10.1016/j.tibs.2016.05.013 27345518

[B3] Bulusu V. Tumanov S. Michalopoulou E. Van Den Broek N. J. Mackay G. Nixon C. (2017). Acetate recapturing by nuclear acetyl-CoA synthetase 2 prevents loss of histone acetylation during oxygen and serum limitation. Cell Rep. 18, 647–658. 10.1016/j.celrep.2016.12.055 28099844 PMC5276806

[B4] Buszewicz D. Archacki R. Palusinski A. Kotlinski M. Fogtman A. Iwanicka-Nowicka R. (2016). HD2C histone deacetylase and a SWI/SNF chromatin remodelling complex interact and both are involved in mediating the heat stress response in Arabidopsis. Plant Cell Environ. 39, 2108–2122. 10.1111/pce.12756 27083783

[B5] Cai L. Sutter B. M. Li B. Tu B. P. (2011). Acetyl-CoA induces cell growth and proliferation by promoting the acetylation of histones at growth genes. Mol. Cell 42, 426–437. 10.1016/j.molcel.2011.05.004 21596309 PMC3109073

[B6] Caretto S. Linsalata V. Colella G. Mita G. Lattanzio V. (2015). Carbon fluxes between primary metabolism and phenolic pathway in plant tissues under stress. Int. J. Mol. Sci. 16, 26378–26394. 10.3390/ijms161125967 26556338 PMC4661826

[B7] Chen C. Li C. Wang Y. Renaud J. Tian G. Kambhampati S. (2017). Cytosolic acetyl-CoA promotes histone acetylation predominantly at H3K27 in *Arabidopsis*. Nat. Plants 3, 814–824. 10.1038/s41477-017-0023-7 28947800

[B8] Chen L. T. Luo M. Wang Y. Y. Wu K. (2010). Involvement of *Arabidopsis* histone deacetylase HDA6 in ABA and salt stress response. J. Exp. Bot. 61, 3345–3353. 10.1093/jxb/erq154 20519338 PMC2905197

[B9] Chen L. T. Wu K. (2010). Role of histone deacetylases HDA6 and HDA19 in ABA and abiotic stress response. Plant Signal. Behav. 5, 1318–1320. 10.4161/psb.5.10.13168 20930557 PMC3115378

[B10] Chen X. Lu L. Mayer K. S. Scalf M. Qian S. Lomax A. (2016). POWERDRESS interacts with HISTONE DEACETYLASE 9 to promote aging in *Arabidopsis*. Elife 5, e17214. 10.7554/eLife.17214 27873573 PMC5119886

[B11] Chen Y. Sprung R. Tang Y. Ball H. Sangras B. Kim S. C. (2007). Lysine propionylation and butyrylation are novel post-translational modifications in histones. Mol. Cell Proteomics 6, 812–819. 10.1074/mcp.M700021-MCP200 17267393 PMC2911958

[B12] Cheng X. Zhang S. Tao W. Zhang X. Liu J. Sun J. (2018). INDETERMINATE SPIKELET1 recruits histone deacetylase and a transcriptional repression complex to regulate rice salt tolerance. Plant Physiol. 178, 824–837. 10.1104/pp.18.00324 30061119 PMC6181036

[B13] De Block M. Verduyn C. De Brouwer D. Cornelissen M. (2005). Poly(ADP-ribose) polymerase in plants affects energy homeostasis, cell death and stress tolerance. Plant J. 41, 95–106. 10.1111/j.1365-313X.2004.02277.x 15610352

[B14] Ding B. Bellizzi Mdel R. Ning Y. Meyers B. C. Wang G. L. (2012). HDT701, a histone H4 deacetylase, negatively regulates plant innate immunity by modulating histone H4 acetylation of defense-related genes in rice. Plant Cell 24, 3783–3794. 10.1105/tpc.112.101972 22968716 PMC3480302

[B15] Earley K. W. Shook M. S. Brower-Toland B. Hicks L. Pikaard C. S. (2007). In vitro specificities of *Arabidopsis* co-activator histone acetyltransferases: implications for histone hyperacetylation in gene activation. Plant J. 52, 615–626. 10.1111/j.1365-313X.2007.03264.x 17877703

[B16] Eremina M. Rozhon W. Yang S. Poppenberger B. (2015). ENO2 activity is required for the development and reproductive success of plants, and is feedback-repressed by AtMBP-1. Plant J. 81, 895–906. 10.1111/tpj.12775 25620024

[B17] Etchegaray J. P. Mostoslavsky R. (2016). Interplay between metabolism and epigenetics: A nuclear adaptation to environmental changes. Mol. Cell 62, 695–711. 10.1016/j.molcel.2016.05.029 27259202 PMC4893201

[B18] Fang H. Liu X. Thorn G. Duan J. Tian L. (2014). Expression analysis of histone acetyltransferases in rice under drought stress. Biochem. Biophys. Res. Commun. 443, 400–405. 10.1016/j.bbrc.2013.11.102 24309107

[B19] Farrona S. Hurtado L. Reyes J. C. (2007). A nucleosome interaction module is required for normal function of Arabidopsis thaliana BRAHMA. J. Mol. Biol. 373, 240–250. 10.1016/j.jmb.2007.07.012 17825834

[B20] Feng J. H. Jing F. B. Fang H. Gu L. C. Xu W. F. (2011). Expression, purification, and S-nitrosylation of recombinant histone deacetylase 8 in *Escherichia coli.* Biosci. Trends 5, 17–22. 10.5582/bst.2011.v5.1.17 21422596

[B21] Gao M. J. Li X. Huang J. Gropp G. M. Gjetvaj B. Lindsay D. L. (2015). SCARECROW-LIKE15 interacts with HISTONE DEACETYLASE19 and is essential for repressing the seed maturation programme. Nat. Commun. 6, 7243. 10.1038/ncomms8243 26129778 PMC4507008

[B22] Han S. K. Wu M. F. Cui S. Wagner D. (2015). Roles and activities of chromatin remodeling ATPases in plants. Plant J. 83, 62–77. 10.1111/tpj.12877 25977075

[B23] Hartl M. Fussl M. Boersema P. J. Jost J. O. Kramer K. Bakirbas A. (2017). Lysine acetylome profiling uncovers novel histone deacetylase substrate proteins in *Arabidopsis*. Mol. Syst. Biol. 13, 949. 10.15252/msb.20177819 29061669 PMC5658702

[B24] Henry E. Fung N. Liu J. Drakakaki G. Coaker G. (2015). Beyond glycolysis: GAPDHs are multi-functional enzymes involved in regulation of ROS, autophagy, and plant immune responses. PLoS Genet. 11, e1005199. 10.1371/journal.pgen.1005199 25918875 PMC4412566

[B25] Henry R. A. Kuo Y. M. Andrews A. J. (2013). Differences in specificity and selectivity between CBP and p300 acetylation of histone H3 and H3/H4. Biochemistry 52, 5746–5759. 10.1021/bi400684q 23862699 PMC3756530

[B26] Hu Z. Song N. Zheng M. Liu X. Liu Z. Xing J. (2015). Histone acetyltransferase GCN5 is essential for heat stress-responsive gene activation and thermotolerance in *Arabidopsis.* Plant J. 84, 1178–1191. 10.1111/tpj.13076 26576681

[B27] Ito K. Hanazawa T. Tomita K. Barnes P. J. Adcock I. M. (2004). Oxidative stress reduces histone deacetylase 2 activity and enhances IL-8 gene expression: role of tyrosine nitration. Biochem. Biophys. Res. Commun. 315, 240–245. 10.1016/j.bbrc.2004.01.046 15013452

[B28] Jin H. Choi S. M. Kang M. J. Yun S. H. Kwon D. J. Noh Y. S. (2018). Salicylic acid-induced transcriptional reprogramming by the HAC-NPR1-TGA histone acetyltransferase complex in *Arabidopsis*. Nucleic Acids Res. 46, 11712–11725. 10.1093/nar/gky847 30239885 PMC6294559

[B29] Kang M. Abdelmageed H. Lee S. Reichert A. Mysore K. S. Allen R. D. (2013). AtMBP-1, an alternative translation product of LOS2, affects abscisic acid responses and is modulated by the E3 ubiquitin ligase AtSAP5. Plant J. 76, 481–493. 10.1111/tpj.12312 23952686

[B30] Kim J. M. Sasaki T. Ueda M. Sako K. Seki M. (2015). Chromatin changes in response to drought, salinity, heat, and cold stresses in plants. Front. Plant Sci. 6, 114. 10.3389/fpls.2015.00114 25784920 PMC4345800

[B31] Kim J. M. To T. K. Matsui A. Tanoi K. Kobayashi N. I. Matsuda F. (2017). Acetate-mediated novel survival strategy against drought in plants. Nat. Plants 3, 17097. 10.1038/nplants.2017.97 28650429

[B32] Kong L. Qiu X. Kang J. Wang Y. Chen H. Huang J. (2017). A phytophthora effector manipulates host histone acetylation and reprograms defense gene expression to promote infection. Curr. Biol. 27, 981–991. 10.1016/j.cub.2017.02.044 28318979

[B33] Krogan N. T. Hogan K. Long J. A. (2012). APETALA2 negatively regulates multiple floral organ identity genes in *Arabidopsis* by recruiting the co-repressor TOPLESS and the histone deacetylase HDA19. Development 139, 4180–4190. 10.1242/dev.085407 23034631 PMC3478687

[B34] Kuo Y. M. Andrews A. J. (2013). Quantitating the specificity and selectivity of Gcn5-mediated acetylation of histone H3. PLoS One 8, e54896. 10.1371/annotation/b2bf9c2e-90a9-4228-9b38-2f1bc977a437 23437046 PMC3578832

[B35] Latrasse D. Jegu T. Li H. De Zelicourt A. Raynaud C. Legras S. (2017). MAPK-triggered chromatin reprogramming by histone deacetylase in plant innate immunity. Genome Biol. 18, 131. 10.1186/s13059-017-1261-8 28683804 PMC5501531

[B36] Lee D. Y. Lee C. I. Lin T. E. Lim S. H. Zhou J. Tseng Y. C. (2012). Role of histone deacetylases in transcription factor regulation and cell cycle modulation in endothelial cells in response to disturbed flow. Proc. Natl. Acad. Sci. U. S. A. 109, 1967–1972. 10.1073/pnas.1121214109 22308472 PMC3277521

[B37] Lee H. Guo Y. Ohta M. Xiong L. Stevenson B. Zhu J. K. (2002). LOS2, a genetic locus required for cold-responsive gene transcription encodes a bi-functional enolase. EMBO J. 21, 2692–2702. 10.1093/emboj/21.11.2692 12032082 PMC126021

[B38] Lee K. W. Chen P. W. Yu S. M. (2014). Metabolic adaptation to sugar/O2 deficiency for anaerobic germination and seedling growth in rice. Plant Cell Environ. 37, 2234–2244. 10.1111/pce.12311 24575721

[B39] Li B. B. Wang X. Tai L. Ma T. T. Shalmani A. Liu W. T. (2018a). NAD kinases: Metabolic targets controlling redox co-enzymes and reducing power partitioning in plant stress and development. Front. Plant Sci. 9, 379. 10.3389/fpls.2018.00379 29662499 PMC5890153

[B40] Li H. Yan S. Zhao L. Tan J. Zhang Q. Gao F. (2014). Histone acetylation associated up-regulation of the cell wall related genes is involved in salt stress induced maize root swelling. BMC Plant Biol. 14, 105. 10.1186/1471-2229-14-105 24758373 PMC4005470

[B41] Li S. Lin Y.J. Wang P. Zhang B. Li M. Chen S., (2019). The AREB1 Transcription Factor Influences Histone Acetylation to Regulate Drought Responses and Tolerance in Populus trichocarpa. Plant Cell 31, 663–686. 10.1105/tpc.18.00437 30538157 PMC6482633

[B42] Li X. Yu W. Qian X. Xia Y. Zheng Y. Lee J. H. (2017). Nucleus-translocated ACSS2 promotes gene transcription for lysosomal biogenesis and autophagy. Mol. Cell 66684–697, e689. 10.1016/j.molcel.2017.04.026 PMC5521213 28552616

[B43] Lin M. Oliver D. J. (2008). The role of acetyl-coenzyme a synthetase in *Arabidopsis*. Plant Physiol. 147, 1822–1829. 10.1104/pp.108.121269 18552233 PMC2492652

[B44] Liu X. Wei W. Zhu W. Su L. Xiong Z. Zhou M. (2017). Histone deacetylase AtSRT1 links metabolic flux and stress response in *Arabidopsis*. Mol. Plant 10, 1510–1522. 10.1016/j.molp.2017.10.010 29107034

[B45] Liu X. Yang S. Zhao M. Luo M. Yu C. W. Chen C. Y. (2014). Transcriptional repression by histone deacetylases in plants. Mol. Plant 7, 764–772. 10.1093/mp/ssu033 24658416

[B46] Long J. A. Ohno C. Smith Z. R. Meyerowitz E. M. (2006). TOPLESS regulates apical embryonic fate in *Arabidopsis*. Science 312, 1520–1523. 10.1126/science.1123841 16763149

[B47] Lu Y. Xu Q. Liu Y. Yu Y. Cheng Z. Y. Zhao Y. (2018). Dynamics and functional interplay of histone lysine butyrylation, crotonylation, and acetylation in rice under starvation and submergence. Genome Biol. 19, 144. 10.1186/s13059-018-1533-y 30253806 PMC6154804

[B48] Luo M. Wang Y. Y. Liu X. Yang S. Lu Q. Cui Y. (2012). HD2C interacts with HDA6 and is involved in ABA and salt stress response in *Arabidopsis*. J. Exp. Bot. 63, 3297–3306. 10.1093/jxb/ers059 22368268 PMC3350937

[B49] Marmorstein R. Zhou M. M. (2014). Writers and readers of histone acetylation: structure, mechanism, and inhibition. Cold Spring Harb. Perspect. Biol. 6, a018762. 10.1101/cshperspect.a018762 24984779 PMC4067988

[B50] Mcdonnell E. Crown S. B. Fox D. B. Kitir B. Ilkayeva O. R. Olsen C. A. (2016). Lipids reprogram metabolism to become a major carbon source for histone acetylation. Cell Rep. 17, 1463–1472. 10.1016/j.celrep.2016.10.012 27806287 PMC5123807

[B51] Mehdi S. Derkacheva M. Ramstrom M. Kralemann L. Bergquist J. Hennig L. (2016). The WD40 domain protein MSI1 functions in a histone deacetylase complex to fine-tune abscisic acid signaling. Plant Cell 28, 42–54. 10.1105/tpc.15.00763 26704384 PMC4746680

[B52] Mengel A. Ageeva A. Georgii E. Bernhardt J. Wu K. Durner J. (2017). Nitric oxide modulates histone acetylation at stress genes by inhibition of histone deacetylases. Plant Physiol. 173, 1434–1452. 10.1104/pp.16.01734 27980017 PMC5291017

[B53] Mews P. Donahue G. Drake A. M. Luczak V. Abel T. Berger S. L. (2017). Acetyl-CoA synthetase regulates histone acetylation and hippocampal memory. Nature 546, 381–386. 10.1038/nature22405 28562591 PMC5505514

[B54] Mutuku J. M. Nose A. (2012). Changes in the contents of metabolites and enzyme activities in rice plants responding to *Rhizoctonia solani* Kuhn infection: activation of glycolysis and connection to phenylpropanoid pathway. Plant Cell Physiol. 53, 1017–1032. 10.1093/pcp/pcs047 22492233

[B55] Nott A. Watson P. M. Robinson J. D. Crepaldi L. Riccio A. (2008). S-Nitrosylation of histone deacetylase 2 induces chromatin remodelling in neurons. Nature 455, 411–415. 10.1038/nature07238 18754010

[B56] Okuda K. Ito A. Uehara T. (2015). Regulation of histone deacetylase 6 activity *via* S-nitrosylation. Biol. Pharm. Bull. 38, 1434–1437. 10.1248/bpb.b15-00364 26328501

[B57] Pandey R. Muller A. Napoli C. A. Selinger D. A. Pikaard C. S. Richards E. J. (2002). Analysis of histone acetyltransferase and histone deacetylase families of *Arabidopsis thaliana* suggests functional diversification of chromatin modification among multicellular eukaryotes. Nucleic Acids Res. 30, 5036–5055. 10.1093/nar/gkf660 12466527 PMC137973

[B58] Park J. Lim C. J. Shen M. Park H. J. Cha J. Y. Iniesto E. (2018). Epigenetic switch from repressive to permissive chromatin in response to cold stress. Proc. Natl. Acad. Sci. U. S. A. 115, E5400–E5409. 10.1073/pnas.1721241115 29784800 PMC6003311

[B59] Patanun O. Ueda M. Itouga M. Kato Y. Utsumi Y. Matsui A. (2016). The histone deacetylase inhibitor suberoylanilide hydroxamic acid alleviates salinity stress in cassava. Front. Plant Sci. 7, 2039. 10.3389/fpls.2016.02039 28119717 PMC5220070

[B60] Pi L. Aichinger E. Van Der Graaff E. Llavata-Peris C. I. Weijers D. Hennig L. (2015). Organizer-derived WOX5 signal maintains root columella stem cells through chromatin-mediated repression of CDF4 expression. Dev. Cell 33, 576–588. 10.1016/j.devcel.2015.04.024 26028217

[B61] Roy D. Paul A. Roy A. Ghosh R. Ganguly P. Chaudhuri S. (2014). Differential acetylation of histone H3 at the regulatory region of OsDREB1b promoter facilitates chromatin remodelling and transcription activation during cold stress. PLoS One 9, e100343. 10.1371/journal.pone.0100343 24940877 PMC4062490

[B62] Ryu H. Cho H. Bae W. Hwang I. (2014). Control of early seedling development by BES1/TPL/HDA19-mediated epigenetic regulation of ABI3. Nat. Commun. 5, 4138. 10.1038/ncomms5138 24938150

[B63] Sabari B. R. Tang Z. Huang H. Yong-Gonzalez V. Molina H. Kong H. E. (2015). Intracellular crotonyl-CoA stimulates transcription through p300-catalyzed histone crotonylation. Mol. Cell 58, 203–215. 10.1016/j.molcel.2015.02.029 25818647 PMC4501262

[B64] Sabari B. R. Zhang D. Allis C. D. Zhao Y. (2017). Metabolic regulation of gene expression through histone acylations. Nat. Rev. Mol. Cell Biol. 18, 90–101. 10.1038/nrm.2016.140 27924077 PMC5320945

[B65] Sadler A. J. Suliman B. A. Yu L. Yuan X. Wang D. Irving A. T. (2015). The acetyltransferase HAT1 moderates the NF-kappaB response by regulating the transcription factor PLZF. Nat. Commun. 6, 6795. 10.1038/ncomms7795 25865065

[B66] Sako K. Kim J. M. Matsui A. Nakamura K. Tanaka M. Kobayashi M. (2016). Ky-2, a histone deacetylase inhibitor, enhances high-salinity stress tolerance in *Arabidopsis thaliana*. Plant Cell Physiol. 57, 776–783. 10.1093/pcp/pcv199 26657894

[B67] Servet C. Conde E Silva N. Zhou D. X. (2010). Histone acetyltransferase AtGCN5/HAG1 is a versatile regulator of developmental and inducible gene expression in *Arabidopsis*. Mol. Plant 3, 670–677. 10.1093/mp/ssq018 20457643

[B68] Shen Y. Issakidis-Bourguet E. Zhou D. X. (2016). Perspectives on the interactions between metabolism, redox, and epigenetics in plants. J. Exp. Bot. 67, 5291–5300. 10.1093/jxb/erw310 27531885

[B69] Shen Y. Lei T. Cui X. Liu X. Zhou S. Zheng Y. (2019). Arabidopsis histone deacetylase HDA15 directly represses plant response to elevated ambient temperature. Plant J. Epub ahead of print 10.1111/tpj.14492 31400169

[B70] Shi L. Tu B. P. (2013). Acetyl-CoA induces transcription of the key G1 cyclin CLN3 to promote entry into the cell division cycle in *Saccharomyces cerevisiae*. Proc. Natl. Acad. Sci. U. S. A. 110, 7318–7323. 10.1073/pnas.1302490110 23589851 PMC3645525

[B71] Song C. P. Agarwal M. Ohta M. Guo Y. Halfter U. Wang P. (2005). Role of an *Arabidopsis* AP2/EREBP-type transcriptional repressor in abscisic acid and drought stress responses. Plant Cell 17, 2384–2396. 10.1105/tpc.105.033043 15994908 PMC1182496

[B72] Sutendra G. Kinnaird A. Dromparis P. Paulin R. Stenson T. H. Haromy A. (2014). A nuclear pyruvate dehydrogenase complex is important for the generation of acetyl-CoA and histone acetylation. Cell 158, 84–97. 10.1016/j.cell.2014.04.046 24995980

[B73] Tan M. Luo H. Lee S. Jin F. Yang J. S. Montellier E. (2011). Identification of 67 histone marks and histone lysine crotonylation as a new type of histone modification. Cell 146, 1016–1028. 10.1016/j.cell.2011.08.008 21925322 PMC3176443

[B74] Tasset C. Singh Yadav A. Sureshkumar S. Singh R. Van Der Woude L. Nekrasov M. (2018). POWERDRESS-mediated histone deacetylation is essential for thermomorphogenesis in Arabidopsis thaliana. PLoS Genet. 14, e1007280. 10.1371/journal.pgen.1007280 29547672 PMC5874081

[B75] Torres E. S. Deal R. B. (2019). The histone variant H2A.Z and chromatin remodeler BRAHMA act coordinately and antagonistically to regulate transcription and nucleosome dynamics in *Arabidopsis*. Plant J. 99, 144–162. 10.1111/tpj.14281 30742338 PMC7259472

[B76] Ueda M. Matsui A. Nakamura T. Abe T. Sunaoshi Y. Shimada H. (2018). Versatility of HDA19-deficiency in increasing the tolerance of *Arabidopsis* to different environmental stresses. Plant Signal. Behav. 13, e1475808. 10.1080/15592324.2018.1475808 30047814 PMC6149488

[B77] Ueda M. Matsui A. Tanaka M. Nakamura T. Abe T. Sako K. (2017). The distinct roles of Class I and II RPD3-like histone deacetylases in salinity stress response. Plant Physiol. 175, 1760–1773. 10.1104/pp.17.01332 29018096 PMC5717743

[B78] Wang L. Wang C. Liu X. Cheng J. Li S. Zhu J. K. (2019). Peroxisomal beta-oxidation regulates histone acetylation and DNA methylation in *Arabidopsis*. Proc. Natl. Acad. Sci. U. S. A. 116, 10576–10585. 10.1073/pnas.1904143116 31064880 PMC6534973

[B79] Wang P. Zhao L. Hou H. Zhang H. Huang Y. Wang Y. (2015). Epigenetic changes are associated with programmed cell death induced by heat stress in seedling leaves of *Zea mays*. Plant Cell Physiol. 56, 965–976. 10.1093/pcp/pcv023 25670712

[B80] Wang Q. J. Sun H. Dong Q. L. Sun T. Y. Jin Z. X. Hao Y. J. (2016). The enhancement of tolerance to salt and cold stresses by modifying the redox state and salicylic acid content *via the* cytosolic malate dehydrogenase gene in transgenic apple plants. Plant Biotechnol. J. 14, 1986–1997. 10.1111/pbi.12556 26923485 PMC5043475

[B81] Wang Y. Hu Q. Wu Z. Wang H. Han S. Jin Y. (2017). HISTONE DEACETYLASE 6 represses pathogen defence responses in *Arabidopsis thaliana*. Plant Cell Environ. 40, 2972–2986. 10.1111/pce.13047 28770584

[B82] Wang Z. Cao H. Sun Y. Li X. Chen F. Carles A. (2013). Arabidopsis paired amphipathic helix proteins SNL1 and SNL2 redundantly regulate primary seed dormancy *via* abscisic acid-ethylene antagonism mediated by histone deacetylation. Plant Cell 25, 149–166. 10.1105/tpc.112.108191 23371947 PMC3584531

[B83] Wellen K. E. Hatzivassiliou G. Sachdeva U. M. Bui T. V. Cross J. R. Thompson C. B. (2009). ATP-citrate lyase links cellular metabolism to histone acetylation. Science 324, 1076–1080. 10.1126/science.1164097 19461003 PMC2746744

[B84] Xie Z. Dai J. Dai L. Tan M. Cheng Z. Wu Y. (2012). Lysine succinylation and lysine malonylation in histones. Mol. Cell Proteomics 11, 100–107. 10.1074/mcp.M111.015875 22389435 PMC3418837

[B85] Yuan L. Liu X. Luo M. Yang S. Wu K. (2013). Involvement of histone modifications in plant abiotic stress responses. J. Integr. Plant Biol. 55, 892–901. 10.1111/jipb.12060 24034164

[B86] Zhang H. Zhao Y. Zhou D. X. (2017). Rice NAD+-dependent histone deacetylase OsSRT1 represses glycolysis and regulates the moonlighting function of GAPDH as a transcriptional activator of glycolytic genes. Nucleic Acids Res. 45, 12241–12255. 10.1093/nar/gkx825 28981755 PMC5716216

[B87] Zhao L. Wang P. Hou H. Zhang H. Wang Y. Yan S. (2014a). Transcriptional regulation of cell cycle genes in response to abiotic stresses correlates with dynamic changes in histone modifications in maize. PLoS One 9, e106070. 10.1371/journal.pone.0106070 25171199 PMC4149478

[B88] Zhao L. Wang P. Yan S. Gao F. Li H. Hou H. (2014b). Promoter-associated histone acetylation is involved in the osmotic stress-induced transcriptional regulation of the maize ZmDREB2A gene. Physiol. Plant 151, 459–467. 10.1111/ppl.12136 24299295

[B89] Zheng M. Liu X. Lin J. Liu X. Wang Z. Xin M. (2018). Histone acetyltransferase GCN5 contributes to cell wall integrity and salt stress tolerance by altering the expression of cellulose synthesis genes. Plant J. 97, 587–602. 10.1111/tpj.14144 30394596

[B90] Zheng Y. Ding Y. Sun X. Xie S. Wang D. Liu X. (2016). Histone deacetylase HDA9 negatively regulates salt and drought stress responsiveness in *Arabidopsis*. J. Exp. Bot. 67, 1703–1713. 10.1093/jxb/erv562 26733691

[B91] Zhou S. Jiang W. Long F. Cheng S. Yang W. Zhao Y. (2017). Rice homeodomain protein WOX11 recruits a histone acetyltransferase complex to establish programs of cell proliferation of crown root meristem. Plant Cell 29, 1088–1104. 10.1105/tpc.16.00908 28487409 PMC5466029

